# Local Use of Chlorate of Potassa in Open Carcinoma

**Published:** 1873-06

**Authors:** 


LOCAL USE OF CHLORATE OF POTASSA IN OPEN CARCINOMA, BY DR. BUROW, SEN.—This remedy has been used by him in five cases of cancerous ulcerations, one twenty-two centimeters in circumference, on the upper arm, three on the mamma, one originating from the periosteum of the upper maxilla and os zygomaticum, which, with the exception of two, had terminated in ulceration without previons operations. It is applied once daily, finely pulverized, over the entire surface of the ulcer. The results of his observations are that the energetic use of chlorate of potassa on these ulcerating cancers produces always diminution and shrinking of the exuberant granulations, resorption of the surrounding infiltrations, lessening of the secretions and of the irritability. He does not consider the value of the remedy established beyond a doubt by his yet limited observations, but calls the attention of the profession to his success, in order to insure further investigation.—*Berliner Klinische Wochenschrift, No.* 6, 1873.

In a later number of the same journal Dr. Dòrger, of Hamburg, states that French physicsans have used the chlorate of potassa ten years ago, according to their statements, with the same effect upon carcinoma as Dr. Burow. The *Union Medical*, 2863, No. 154, contains the results of the observations of Bergeron, Milon, and Blondeau, on the treatment of carcinoma by chlorate of potassa. They used very strong or concentrated solutions of this salt locally, and obtained cures within a few months. Debont's (*Bull de Thix*, lxvi, page 12, January 25, 1864; *Schmidt's Jahrbücher*, 1865, vol. i, page 170) and besides him Lebbane's, Cook's, Charcot's, Delpeche's, and Wishon's observations coincide fully with those of Dr. Burow. (The conformity of these therapeutical of results of so many makes further trials in the interest of the sufferers with this dreadful malady highly desirable.)—*Berliner Klinische Wochenscrift, No.* 12, 1873.

